# Mechanism of modulation through PI3K-AKT pathway about *Nepeta cataria* L.’s extract in non-small cell lung cancer

**DOI:** 10.18632/oncotarget.15608

**Published:** 2017-02-22

**Authors:** Jiaxin Fan, Yongrui Bao, Xiansheng Meng, Shuai Wang, Tianjiao Li, Xin Chang, Guanlin Yang, Tao Bo

**Affiliations:** ^1^ School of Pharmacy, Liaoning University of Traditional Chinese Medicine, Dalian 116600, P. R. China; ^2^ Component Medicine Engineering Research Center of Liaoning Province, Dalian 116600, P. R. China; ^3^ Liaoning Province Modern Chinese Medicine Research Engineering Laboratory, Dalian 116600, P. R. China; ^4^ Liaoning University of Traditional Chinese Medicine-Agilent Technologies Modern TCM and Multi-Omics Research Collaboration Lab, Dalian 116600, P. R. China

**Keywords:** non-small cell lung cancer, A549 cell, MicroRNA-126, inhibition of proliferation, PI3K-AKT signaling pathway

## Abstract

Non-small cell lung cancer (NSCLC) is regarded as one of the major intractable diseases, which was cured mainly by chemotherapeutics in the clinical treatment at present. But it is still a vital mission for the current medical and researchers that hunting a natural medicine which have little side effects and high-efficiency against the NSCLC on account of the shortcomings on current drugs. *Nepeta cataria* L. plays an important role in anti-cancer treatment according to the reports which was recorded in the Chinese Pharmacopoeia of version 2015 and belongs to one of the Traditional Chinese medicine (TCM). Microfluidic chip technology is widely used in scientific research field due to its high-throughput, high sensitivity and low cost with the continuous progress of science and technology. In this study, we investigate the effect of total flavonoid extracted from *Nepeta cataria* L. (TFS) through human lung cancer cell line A549 based on the microfluidic device and Flow Cytometry. So we detected the mRNA expression of MicroRNA-126 (miR-126), VEGF, PI3K, PTEN and proteins expression respectively to explore the partial PI3K-AKT pathway molecular mechanisms through Quantitative Real-time PCR (qRT-PCR) and Western Blot. The results showed that TFS can disturb the expression of miR-126 and regulate the PI3K-AKT signaling pathway to meet the effect of anti-cancer. Taking all these results into consideration we can draw a conclusion that TFS may be used as a novel therapeutic agent for NSCLC in the near future.

## INTRODUCTION

Lung cancer is one of the major diseases which were seriously endangering human's health and life according to related data statistics, the death rate of cancer presents an increasing trend year by year, especially NSCLC which was the most common type of lung cancer. At present, the method with chemotherapeutics was popular and efficacious in the treatment of NSCLC because it can quickly kill the cancer cells. But with the extension of the treatment time, NSCLC cells tend to be resistant to such treatments, contributing to the local recurrence of NSCLC [1]. In summary, drug resistance and cytotoxicity remains the major therapeutic challenge in current clinical practice [2], so seeking a high efficiency and low toxicity drug from our natural world is beneficial to the entire social development of human beings. In resent years, many researchers whose focus shifted from the chemotherapeutics to the TCM aimed to find a novel targeted drug for lung tumor therapy. It is well known that TCM has attracted the majority scholars’ interest through its numerous advantages, including abundant resources, ancient application history and higher clinical effect. In brief, TCM will have a good potential application in cancer treatment. *Nepeta cataria* L. (family, Limiaceae; order, Lamiales), is one of the TCM which was found in the Eastern Mediterranean, Southern Asia and China [3–5]. It is distributed all round the world, for example north, northwest, northeast and west, commonly used in local people's daily life. Most of the people regard it as antipyretic, antispasmodic, sedative, diuretic and diaphoretic. Volatile oil, flavonoid and lactones are the major effective components, and the content is high. From the literature investigation we can easily see that the volatile oil and flavonoid play important roles in many pharmacological actions, such as anti-cancer including breast cancer and prostatic cancer. So *Nepeta cataria* L. is a promising drug for anti-cancer agent. We have confirmed the most ingredients of TFS, and the chemical compositions which including Icynaroside, Luteolin, Apigenin, Hesperidin and Diosmetin has been accepted in the Journal of Acta Pharmaceutica Sinica in our previous study [6]. But so far the mechanism of anti-cancer action has not been understood absolutely.

We all know that miRNAs plays an important role in many process among our body according to the investigations. miRNAs is a class of small RNAs (18-25 nt) that negatively regulate gene expression by connecting to the 3′-untranslated region (3′-UTR) of their target gene mRNAs [7]. More and more studies show that miRNAs has a close ties with cancer. Such as miR-126, a small regulatory RNAs, which was also known as miR-126- 3p [8]. Its existence may regulate several important biological processes, including cell proliferation, development, apoptosis and epigenetic changes [9, 10], so this portion has become an important part of drug research from explore the activity of miRNAs to explore the mechanism of anti-tumor effect. PI3K-AKT signaling pathway has already been found to be associated with cancer. A study by Luan *et al* demonstrated that overexpression of miR-126 suppressed PI3K and AKT activation in malignant glioma [11, 12]. The decrease of miR-126 in lung cancer has a closest connection to the prognosis. Chen *et al* and Wang *et al* both used qRT-PCR to evaluate the expression of miR-126 between cancer cells and adjacent normal tissues *in vivo* [13–15]. All these researches indicated that miR-126 plays a significant role in the treatment of lung cancer through the PI3K-AKT signaling pathway, at the same time, finding a appropriate clinical drug based on the traditional applications from mechanism research has become an important method of the drug discovery. In our current research, some related technologies including molecular biology technology and microfluidic chip technology were adopted to study TFS's mechanism in treatment of NSCLC with the purpose of laying a foundation for developing anti-cancer drug.

## RESULTS

### Growth activity of cell in chip

**Figure 1:**
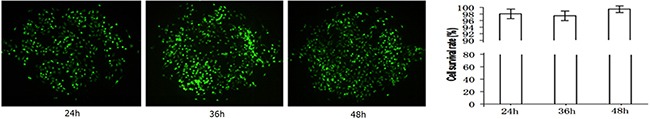
The results of cell viability test (×40) AO can across the intact cell membrane in order to combined with DNA emits intense green fluorescence; EB can across the broken cell membrane in order to combined with DNA emits intense orange-red fluorescence. All the cells were count by the Imageproplus (IPP, version 6.0) for calculating the Cell survival rate (%). The Cell survival rate (%) = (normal cells / the total number of A549 cells) ×100%.

We created a microfluidic culture device to test the cell viability of A549 with the reagents of AO/EB. As we all know that the living cells can be stained green while the dead were red. In this experiment, as seen in Figure 1A, the cells were cultured in chip for 24 h, 36 h, and 48 h. We can see that green fluorescence was accounted for the majority of the area from these 3 pictures. Experimentally, the cell survival rate (%) can achieve 98%. The chart of cell survival rate in chip was seen in Figure 1B. The result illustrates that PDMS has no cell toxic and side effects and the design of this chip is successful for cell culture. In a word, this microfluidic culture device can provide stable and beneficial growth environment for the subsequent experiments.

### Effect of TFS on proliferation of A549 cells

**Figure 2:**
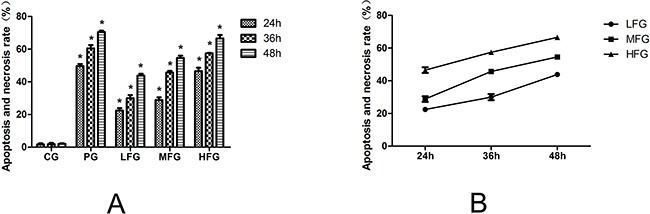
The effect of TFS on the proliferation of A549 cells The Hochest33342 and PI staining Assay used to detect the effect on the proliferation of A549 Cells. The apoptosis and necrosis rate was display in Figure 3A, *P<0.01vs CG. CG represents control group; PG represents positive group; LFG represents low total flavonoid extracted from *Nepeta cataria* L. group; MFG represents middle total flavonoid extracted from Nepeta cataria L. group; HFG represents high total flavonoid extracted from *Nepeta cataria* L. group; From the Figure 3B, we can see the variation tendency of the Apoptosis and necrosis rate.

The Hochest33342 and PI staining solution were used to detect the proliferation of A549 cells after drug stimulating. The result was displayed in Figure 2 which indicated that TFS has a significant anti-tumor effect in lung cancer cell line. In Figure 2A, we can easily see that the apoptosis and necrosis rate of A549 cells in administration group was higher than those in control group significantly (*P*<0.01). In addition, the apoptosis and necrosis rate was increased along with the change in drug concentration and stimulating duration gradually. In short, this phenomenon exhibits a positive correlation on time-effect and concentration-response as seen in Figure 2B. The result showed that TFS has a clinical potential value for the research of anti-cancer.

### MiR-126 expression in A549 cells

**Figure 3:**
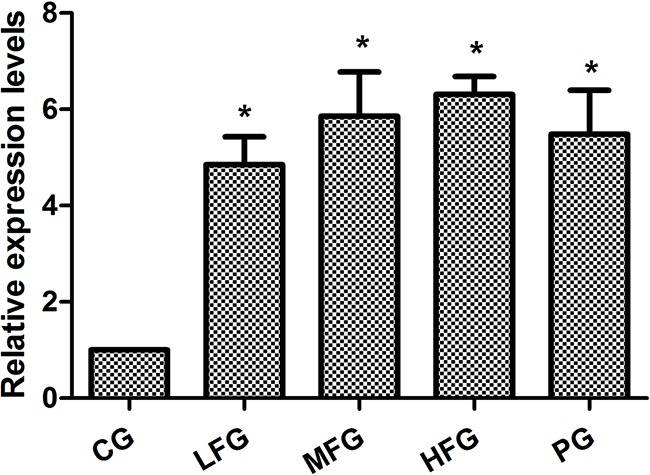
The expression of miR-126 in human lung cancer cell line A549 (mean+/−SEM, n=3) *P<0.01vs CG, *P*<0.05 as significant difference.

qRT-PCR was used to detect miR-126 expression in the human lung cancer cell line A549 including CG, PG and administration group (LFG, MFG and HFG). The results displayed that the relative expression levels of miR-126 compared with U6 in administration group and PG were remarkably higher than those in the CG (*P* < 0.01; Figure 3).

### Cell apoptosis by Flow Cytometry

**Figure 4:**
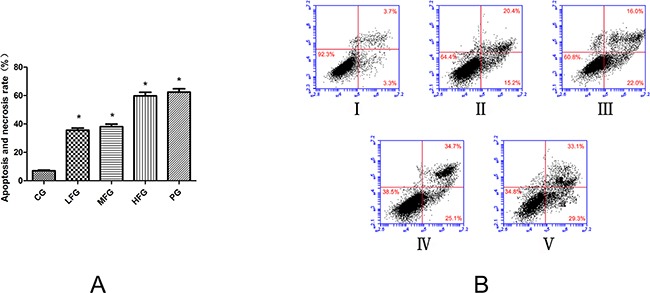
The pro-apoptotic effect of TFS on lung cancer cells (mean+/−SEM, n=3) Cell apoptosis profiles by Annexin V-FITC/PI double fluorescent assay of A549 cells through flow cytometry. In Figure 5A, it is the histogram of Apoptosis and necrosis rate in each group. They all treated with TFS and Cisplatin in different concentrations for 36 h. In Figure 5B, I represents CG, II represents LFG, III represents MFG, IV represents HFG, V represents PG. *compared with CG, *P*<0.05 as significant difference.

The impact of TFS induced A549 cells apoptosis was determined by an Annexin V-FITC/PI double fluorescent assay. From the statistical data we can easily know that different does of TFS have different level effects on pro-apoptotic. The apoptosis and necrosis rate in low dose group of TFS (LFG) was (35.6 ± 1.5) % (*P* <0.01, vs. CG). Middle dose group of TFS (MFG) was (38.0 ± 1.9) % (*P* <0.01, vs. CG). High dose group of TFS (HFG) was (59.8 ± 2.5) % (*P* <0.01, vs. CG). But what should be noticed was that the apoptosis and necrosis rate in positive group (PG) was close to the HFG, is (62.4 ± 2.4) %, all these data was shown in Figure 4.

### The effect of TFS on VEGF, PI3K, PTEN mRNA and protein expression in A549 cells

**Figure 5:**
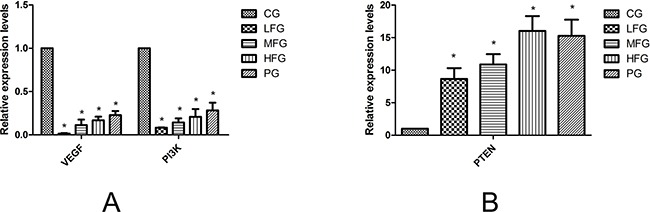
The quantitative real-time polymerase chain reaction in A549 cells The mRNA expressions of VEGF and PI3K was in the Figure 6A, The PTEN mRNA expressions was in Figure 6B, from the picture, we can see that VEGF and PI3K wassignificantly decreased while the PTEN was increased in admintration groups compared with CG. *compared with CG, *P*<0.05 as significant difference.

**Figure 6:**
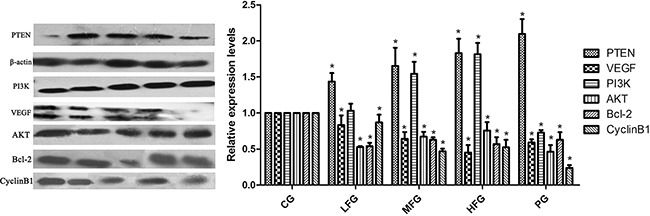
The protein expressions in PTEN/PI3K/AKT signaling pathway CG represents control group; PG represents positive group; LFG represents low total flavonoid extracted from *Nepeta cataria* L. group; MFG represents middle total flavonoid extracted from *Nepeta cataria* L. group; HFG represents high total flavonoid extracted from *Nepeta cataria* L. group; *compared with CG, *P*<0.05 as significant difference.

**Figure 7:**
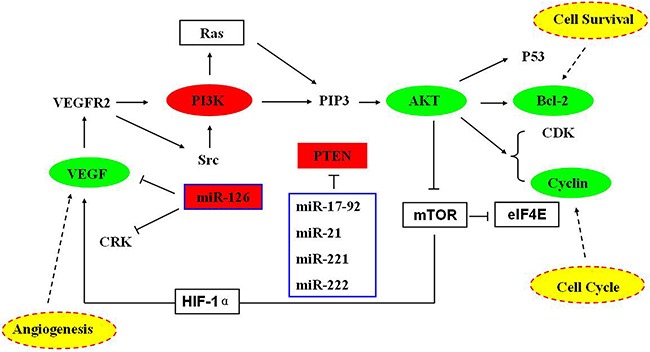
The signaling pathways of TFS anti-NSCLC cancer Different color shows different meanings: Red represents this protein upregulated, Green represents this protein downregulated, Yellow represents the proteins function.

qRT-PCR and Western blot were used to detect the expression of VEGF, PI3K, PTEN in the human lung cancer cell line A549 with related kits. The data obtained from qRT-PCR were analyzed by 2^−CT^. The results suggested the expresssion of VEGF, PI3K in administration group were lower than that in CG (*P* < 0.05, Figure 5A), and the PTEN was overexpression (*P* < 0.05, Figure 5B). The results of western blot suggested that, compared with those in CG, the PTEN, PI3K protein levels were remarkably upregulated and the VEGF, AKT, Bcl-2, CyclinB1 expression downregulated in administration group (*P* < 0.05, Figure 6). All the results from this experiment can come to a conclusion that the TFS has a function on anti-lung cancer through regulating the PTEN/PI3K/AKT signaling pathway. The specific signaling pathway can be seen in Figure 7.

## CONCLUSION

TFS, as a group of compounds extracted from *Nepeta cataria* L., which has higher antitumor activity from previous researches [16–19], and the composition of TFS has been testified involving six compounds, including Diosmetin, Apigenin, Luteolin, Hesperidin, Icynaroside, Quercitrin. We can come to a conclusion that TFS has the function of proliferation-inhibition and pro-apoptosis on lung cancer cells A549 with the help of microfluidic chip device from the current data results. Furthermore, it can also regulate the mRNA expression including miR-126, VEGF, PTEN and PI3K, protein expression including VEGF, PTEN, AKT, Bcl-2, CyclinB1 and PI3K. In a world, all the data might provide that TFS has clear chemical composition and mechanism of anti-cancer action. It has the potential to be developed into a new anti-NSCLC drug from the view of molecular level.

## DISCUSSION

As we all know that the technology of microfluidic chip has the significant advantages and great potentialities for the development of TCM. In this research, we firstly used this microfluidic chip for the related research about *Nepeta cataria* L. On the one hand, this chip used in this research has four culture chambers in each channel, and this design comes from the repeated test, so we can get everything done at one time to avoid duplication of operating and enlarge operating error in this chip. At the same time, injection pump, which was regarded as the powerful source of motivation, can reduce the consumption of reagents under the flow rate of 0.2 μL·min^−1^. And the total consumption of related reagents didn't reach 0.6 mL after treatment for 48 h. It is superior to traditional technology which seeds the cells into 96-well plates. On the other hand, the microfluidic system can provide an experimental condition which was similar to the human body microenvironment [20–23].

In this article, we can easily see that TFS has a high-efficiency for NSCLC cancer, because the proliferation inhibition in HFG was (57.41 ± 0.3)%, (60.48 ± 2.0)% in PG when administrated 36 hours, and it doesn't has significant difference. So the TFS has the same anti-NSCLC cancer ability compared with Cisplatin. Additionally, researches have discovered many signaling pathways which possess certain internal relations with the lung cancer, such as PI3K-AKT signaling pathway (Map 04151 in KEGG) [24–26], VEGF signaling pathway (Map 04370 in KEGG) [27–29], P53 signaling pathway (Map 05223 in KEGG) [30–32] and so on. Among these pathways, it is a common consensus that the PTEN is not only a suppressor gene but also a dual proein/lipid phosphatase. The main substrate is phosphatidylinositol (3,4,5) triphosphate (PIP3), which is the product of PI3K [33]. The protein of Bcl-2 and Cyclins, which have a close correlation with the cell apoptosis and cell cycles respectively. VEGF, an important mediator of blood vessel growth, repress endothelial cell proliferation and migration, survival, and also angiogenesis, a process that facilitates cure the neoplastic diseases. In the present study, we used the qPCR to detect the relative expression level of miR-126, VEGF, PI3K and PTEN, western blot to detect the protein relative expression level of VEGF, PI3K, PTEN, AKT, Bcl-2 and CyclinB1 to make up the vacancy. The result was shown in Figure 3, Figure 5 and Figure 6. From the picture we can easily see that TFS downregulated the mRNA levels of VEGF, PI3K, upregulated the mRNA levels of PTEN. The change trend of protein expression was similar to the result of mRNA's expression. The overexpression of miR-126 impaired cell proliferation in NSCLC A549 cells through regulation of the PI3K/AKT signaling pathway [34–35]. miR-126, a small regulatory RNA whose existence may regulate several biological processes, including cell proliferation, development, apoptosis and epigenetic changes, and it has been confirmed correctly in KEGG reference pathway about microRNAs in cancer. It is surprising that the PI3K was overexpression. So we can speculate the effect of TFS may regulate PI3K/AKT signaling pathway through the PTEN's branch not the PI3K's branch. Taking all these results into consideration, we can draw a conclusion that TFS may be a new anti-lung cancer drug in the future.

## MATERIALS AND METHODS

### Microfluidic chip fabrication

**Figure 8:**
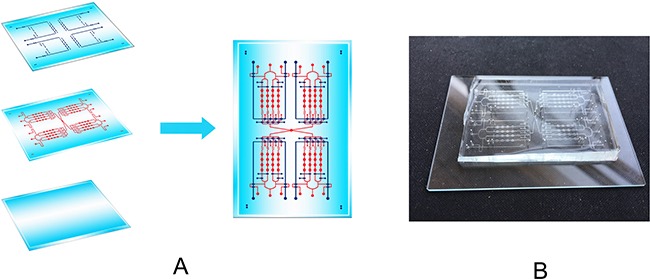
The design of a cell culture microfluidic chip **(A)** The schematic design of the microfluidic chip with VCL and cell culture chambers. The channel of blue was the VCL including GLs and LLs, the red channels was FCL, the function of the ellipses was for cells growth. **(B)** The pictorial diagram of the chip.

The schematic design of microfluidic device contains two layers of Polydimethylsiloxane (PDMS, Dow Corning, Midland, MI, USA). The first layer, we regarded it as the Valve Control Layer (VCL) including Gas valves (GLs) and Liquid valves (LLs), the second layer was regarded as Fluid Channel Layer (FCL), the last layer was the glass for cell culture. All these three layers were combined together by the oxygen plasma for irreversible chemical bonding. The schematic diagram of this chip was shown in Figure 8A.

The chip was manufactured by standard soft lithography method [36]. At first, silicon template and glass were all cleaned follow by acetone, ethanol and water for 30 min altogether, after this, blowdown the water on their surfaces by man-made airflow, and put them in a 105°C heating-platform to make them completely dry. The dried silicon was prepared by spin-coating a layer of SU8-2075 negative photoresist (Microchem, Newton, MA, USA) and patterned by photolithography. After those processes including pre-bake, mid-bake, af-bake, the master model with pattern we designed was got. Secondly, the VCL, which has four LLs and sixteen GLs, was fabricated with PDMS and Hardener (Sylgard 184, Dow Corning, Midland, MI, USA) at a ratio of 8:1 in mass, and the FCL was fabricated at a ratio of 15:1 in mass (PDMS:Hardener). All the mixture was degassed under vacuum in reduced pressure oven before pouring on to the master model. Finally, the main differentiation between VCL and FCL was the mode of their forming method. VCL was using the method of injection molding, FCL was spin-coating.

The elliptical configuration, which was 1.5 mm (length)×1.0 mm (width)×0.05 mm (height) in the VCL, plays an important part in valve control. None-controlled branches were close to 0.1 mm (width). There are also 4×4 columns of cell culture units with four oval-shape modules in the FCL, Each cell culture module was close to 1.0 mm (length)×0.5 mm (width)×0.05 mm (height). The part of fluid channel was 0.2 mm (width). A stereomicroscope was used to ensure the optimal combine of the two layers. The polymer was oven-cured for 40 min at 65°C. After cooling, the PDMS layer was gently peeled from the master and trimmed to size. The resulting PDMS structures were oxidized in oxygen plasma for 3 min for irreversible chemical bonding to glass slides [37]. The pictorial diagram of the chip was displayed in Figure 8B.

### Cell culture

The human lung cancer cell line A549 was purchased from the Cell Bank of Type Culture Collection of Chinese Academy of Sciences (Shanghai, China). It was incubated in Roswell Park Memorial Institute-1640 medium (RPMI 1640, NIBCO, Grand Island, USA) containing 10% fetal bovine serum (FBS, Grand Island, USA) in a carbon dioxide incubator (Nuaire, USA) with 5% carbon dioxide and humidity of 95% at 37°C. When the cells were grown in a monolayer and the adherence rate was approximate 80%, the cells were digested with 0.25% pancreatin (Trypsin-EDTA, Gibco, Grand Island, USA) for subculture process, the solution were transformed into single-cell suspensions by blowing and beating in RPMI-1640. Before cells inoculation, the chip was filled with poly-l-lysine solution (0.01%, m/v) (Sigma–Aldrich, St. Louis, MO, USA) for 1 h to coat their inner surface and increase the adherence rate of cells. Then, the suspensions were injected into the chip for dynamic culture by peristaltic injection pump (Longer Pump, LSP04-1A, China).

### Growth activity of cell in chip

As we all known that the dye of Acridine orange (AO) and Ethidium bromide (EB) has the function on detecting the activity of cell. On the basis of these effects, Lung cancer cell line A549 was cultured 24h, 36h, 48h in the chip respectively. AO and EB were mixed in the dark according to ratio of 1: 1 (v/v). The mixture was injected into the area of cell culture in chip through precision syringe, staining 5 min at room temperature in the dark, and then, washed 3 times in phosphate-buffered saline (1×PBS). The chip was taken into the Inverted Fluorescence Microscope (Nikon ECLIPSE TI, Nikon, Japan) for photos after these complicated operations to calculate the Cell survival rate (%).

### Extract preparation

Sample preparation was performed as follow: Weigh 50 g Schizonepeta herb's powder (40 mesh sieve) accurately in a 1000 mL round-bottom flask, and add 15 times 75 % ethanol solution to reflux extraction 3 times (1 hour per time), filtered, the filtrates were combined together. In addition, adjust the terminal concentration of this drug to 0.1 g·mL^−1^ (m/v). After this, HPD-400 macroporous resin was used to purify the crude extract for two times, collecting the filtrate, heating in water bath around 60°C. The extraction of *Nepeta cataria* L. can be obtained.

### Cell treatment

The single cell suspensions will be inoculated in the chip for long-term culture *in vitro* when they were in the logarithmic growth phase. Under normal circumstances, most of the A549 cells will adhere to the chip within four hours. After that, a certain concentration of TFS (Lab-made, Purity>60%, 0.25 mg·mL^−1^ as the Low-dose group (LFG), 0.5 mg·mL^−1^ as the Middle-dose group (MFG), 1.0 mg·mL^−1^ as the High-dose group (HFG)) were injected into the chip via a precision syringe at a flow rate of 0.2 μL·min^−1^ for dynamic administration. After treated 24h, 36h, 48h respectively, the cells were incubated for 10 min with mixture dye including Hoechst 33342 and propidium iodide (PI) in a volume ratio of 1: 1 in dark. After this operation, Inverted fluorescence microscope (Nikon ECLIPSE TI, Nikon, Japan) was used to take pictures of cells for calculating the apoptosis and necrosis rate (%).

### Analysis of cell apoptosis by flow cytometry

The single-cell suspensions were also injected into the 6-well plates. 0.0, 0.25, 0.5, 1.0 mg·mL^−1^ of TFS and a positive control drug (PG, Cisplatin, 10 μg·mL^−1^, He pharmaceutical co., LTD., Jiangsu, China) were added into the plates for stimulating 48 h while the adherence rate was approximate to 70%. After this operation, the percentages of apoptotic cells were determined by Annexin V-FITC Apoptosis Detection Kit (Vazyme biotech, Nanjing, China) following the manufacturer's recommendation. 400 μL 1×Binding Buffer was added to each group after cells were incubated in the dark for 10 min with 5 μL Annexin V-FITC and 5 μL PI. After that operation, the analysis was executed through the Flow Cytometry (BD Accuir C6, USA).

### Quantitative real time PCR

Total RNA in each group was isolated from lung cancer cell (A549) using SanPrep Column microRNA Mini-Preps Kit (Sangon Biotech, China) following the manufacturer's recommendation [38]. The purity and concentration of the total RNA was detected by the optical density (OD) at 260 nm/280 nm in ultraviolet spectrophotometer. On the one hand, the miR-126 was reverse transcribed using miRNA First Strand cDNA Synthesis Kit (Sangon Biotech, China), Stem-loop qRT-PCR was performed using the MicroRNAs Quantitation PCR Kit (Sangon Biotech, China) with Piko Thermal Cycler 96-well system (Piko, Hawaii State, USA) according to the manufacturer's protocol. U6 small nuclear RNA was used as the internal reference. Relative expression was evaluated by the comparative Ct method and normalized to the expression of U6 small nuclear RNA [5]. On the other hand, the detection of PTEN, VEGF and PI3K reverse transcription was carried out using the First-strand cDNA Synthesis Kit (TransGen Biotech, China) following the manufacturer's recommendation. qRT-PCR was performed by the *TransStart* Top Green qPCR SuperMix (TransGen Biotech, China) with Piko Thermal Cycler 96-well system. The β-actin mRNA was employed as an internal control. The relative quantification of mRNA expression was also determined using the comparative Ct method. The reaction conditions were as follows: pre-denatured at 95°C for 10 minutes, denatured at 95°C for 10 seconds, annealed at 60°C for 20 seconds, and extended at 72°C for 34 seconds, with a total of 40 cycles [5]. All of the groups were analyzed in triplicate independent experiments. The amplified primer sequences of miR-126, reference U6 and related Gene are listed in Table 1.

**Table 1:** Primer sequences

Gene	Primer Sequence
miR-126	Forward: 5′-TGGCGGTCGTACCGTGAGTAAT-3′
	Reverse: 5′-ATCCAGTGCAGGGTCCGAGG-3′
U6	Forward: 5′-AGAGAAGATTAGCATGGCCCCTG-3′
	Reverse: 5′-ATCCAGTGCAGGGTCCGAGG-3′
β-actin	Forward: 5′-CACCCGCGAGTACAACCTTC-3′
	Reverse: 5′-CCCATACCCACCATCACACC-3′
VEGF	Forward: 5′-AGGAGTACCCTGATGAGATCGAGTA-3′
	Reverse: 5′-TGGTGAGGTTTGATCCGCATA-3′
PTEN	Forward: 5′-CCCAGTCAGAGGCGCTATG-3′
	Reverse: 5′-GGCAGACCACAAACTGAGGATT-3′
PI3K	Forward: 5′-AACGAGAACGTGTGCCATTTG-3′
	Reverse: 5′-AGAGATTGGCATGCTGTCGAA-3′

### Western blotting

Cells were homogenized in RIPA buffer containing 1 mM PMSF (Cell-Signaling Tech., US). The protein concentrations were determined by a Lowry Kit (Solarbio, Beijing, China). Equal amount of proteins were separated on 10% SDS-PAGE and then transferred into polyvinylidene fluoride (PVDF) membrane (Bio-Rad, US). After this, the membranes were blocked in 5% bovine serum albumin (BSA, Solarbio, Beijing, China) for shaking 2 h at room temperature, 1×TBST buffer was used to wash the membranes for 3 times (10 min per time). Subsequently, the membranes incubated overnight at 4°C with the following primary antibodies: anti-PTEN (1:1000, Cell-signaling Tech.), anti-AKT (1:1000, Cell-signaling Tech.), anti-Bcl-2 (1:1000, Cell-signaling Tech.), anti-CyclinB1 (1:1000, Cell-signaling Tech.), anti-PI3 Kinase p85(1:1000, Cell-signaling Tech.), anti-VEGF (1:1000, Abcam, Santa Cruz, USA) and anti-β-actin (1:1000, Cell-signaling Tech.), which served as an internal reference control. Next, the membranes incubated with horseradish peroxides-conjugated secondary antibody (goat anti-rabbit IG (1:5000) or goat anti-mouse IgG (1:5000); Cell-signaling Tech.) for 2 h at room temperature. The membrane was washed 3 times with 1×TBST buffer (10 minutes per time). At last, the antigen-antibody complexes were visualized through the chemilucent ECL (TransGen Biotech, China) detection system. The blots were scanned and analyzed through the IPP software (version 6.0).

### Statistical analysis

All continuous variables were processed using SPSS software (version 19.0) and presented as the mean ± standard deviation (SD). The least significance test was conducted through the analysis of variance (ANOVA) followed by a post-hoc t-test between the groups. Differences in the mean with *P* < 0.05 were considered statistically significant.
